# Research on the Algorithm of Position Correction for High-Speed Moving Express Packages Based on Traditional Vision and AI Vision

**DOI:** 10.3390/s24030892

**Published:** 2024-01-30

**Authors:** Ning Dai, Zhehao Lu, Jingchao Chen, Kaixin Xu, Xudong Hu, Yanhong Yuan

**Affiliations:** Key Laboratory of Modern Textile Machinery & Technology of Zhejiang Province, Zhejiang Sci-Tech University, Hangzhou 310018, China

**Keywords:** package positioning, combination of traditional vision and AI vision, YOLOv5, label allocation

## Abstract

The rapid development of the logistics industry poses significant challenges to the sorting work within this sector. The fast and precise identification of moving express parcels holds immense significance for the performance of logistics sorting systems. This paper proposes a motion express parcel positioning algorithm that combines traditional vision and AI-based vision. In the traditional vision aspect, we employ a brightness-based traditional visual parcel detection algorithm. In the AI vision aspect, we introduce a Convolutional Block Attention Module (CBAM) and Focal-EIoU to enhance YOLOv5, improving the model’s recall rate and robustness. Additionally, we adopt an Optimal Transport Assignment (OTA) label assignment strategy to provide a training dataset based on global optimality for the model training phase. Our experimental results demonstrate that our modified AI model surpasses traditional algorithms in both parcel recognition accuracy and inference speed. The combined approach of traditional vision and AI vision in the motion express parcel positioning algorithm proves applicable for practical logistics sorting systems.

## 1. Introduction

With the exponential growth of the e-commerce sector, logistics companies are encountering a significant surge in order volumes and an intensified demand for sorting tasks [1,2,3,4]. Currently, conventional manual sorting methods are inadequate to manage these escalated sorting demands. Confronted with an increasing volume of parcels, logistics companies are compelled to efficiently execute the sorting of these items. Recently, the adoption of intelligent sorting systems for the efficient sorting of parcels during transportation has emerged as a strategic solution among logistics companies [5]. Research in the realm of intelligent logistics is increasingly prevalent [6,7,8,9,10], encompassing diverse areas, including mechanical structures and electronic control systems. These investigations include theoretical, simulation-based, and experimental facets, substantially improving the efficiency and precision of parcel sorting. These studies clearly indicate that the cornerstone of intelligent logistics sorting is the accurate identification of parcels. Consequently, the investigation into vision-based intelligent logistics sorting systems holds utmost importance.

**Figure 1:**
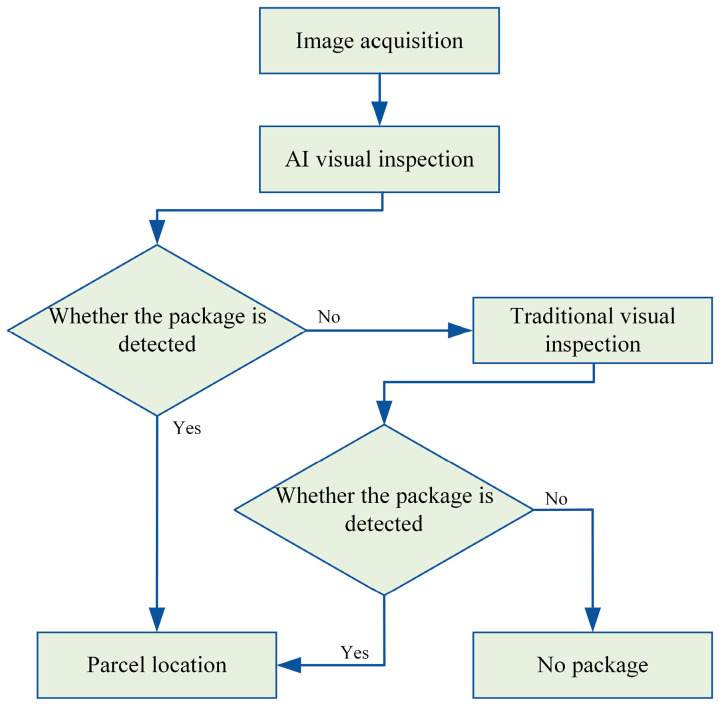
Motion express package positioning algorithm.

In intelligent sorting systems, the accurate positioning of moving packages is a critical factor in determining the successful continuation of the sorting process, and it also plays a pivotal role in enhancing the efficiency of intelligent logistics sorting systems. Building upon this, the paper primarily focuses on pinpointing the location of moving parcels on conveyor trolleys and relaying the identified coordinates to the intelligent logistics sorting system, thus enabling the adjustment of parcel placement on the trolley. Employing solely traditional vision techniques for package identification is significantly susceptible to environmental disturbances, leading to potential misidentification of elements such as dirt and oil stains as packages. Conversely, exclusive reliance on AI technology for package identification could result in overlooking rarer and irregularly shaped parcels. To address these challenges, the approach integrates both traditional vision techniques, leveraging brightness values, and an AI algorithm grounded in YOLOv5 to ascertain the location of express parcels on moving trolleys. Upon acquiring the package image, the AI algorithm is initially deployed for detection. If detection is successful, the package’s offset relative to the trolley is transmitted to the main control system. In the event of non-detection by the AI algorithm, traditional visual inspection methods are employed. This serves as a supplementary diagnostic procedure. Should both tests fail to detect a package, it is concluded that no package is present on the trolley. This method significantly improves the accuracy of parcel sorting within intelligent logistics systems. Figure 1 illustrates the algorithm for locating moving express parcels in the intelligent logistics system.

The principal contributions of this study are as follows:

1. Development of an enhanced network architecture based on YOLOv5, aimed at augmenting the model’s proficiency in detecting and localizing moving express parcels.
2. This study introduces a novel algorithm for positioning moving express parcels, integrating traditional vision techniques based on brightness values with AI-driven vision.

## 2. Related Work

To enhance the sorting efficiency and accuracy in intelligent logistics sorting systems, numerous researchers have integrated visual technology into these systems, employing methodologies like image processing and machine learning to facilitate automatic detection, code scanning, and sorting of express parcels. Researchers worldwide have conducted comprehensive studies on visual technology within intelligent logistics sorting systems, yielding significant outcomes. This section critically reviews these scholarly works and summarizes the key findings.

Kim [11] conducted research on parcel box recognition, using artificial intelligence deep learning technology. The YOLOv5 model was employed to achieve box detection and position estimation, and the model demonstrated relatively fast capabilities in parcel box recognition. Xu [12] proposed a deep learning and multi-information fusion express recognition method, utilizing deep learning to detect targets and employing the ZBAR algorithm for decoding captured barcode images, thus realizing parcel sorting. Han [13] proposed a robot sorting method based on multitask deep learning to achieve accurate detection and efficient sorting of randomly piled express parcels. The accuracy and real-time performance of this method were verified through robot sorting experiments. Wu Cuiling [14] designed an improved Faster RCNN network model to achieve faster recognition and tracking of express parcels. Zhao [15] proposed an improved Faster R-CNN parcel detection method, addressing various degrees of false detection in existing parcel detection methods. N. Ladplee [16] presented an automated volume measurement system for rectangular parcel boxes based on a single LiDAR depth camera. This system extracted the width, length, and height of the boxes, with an average processing time of 1.01 s. X. Duan [17] investigated express parcel detection and shape recognition in a single camera setup, reducing the cost of express parcel recognition. Vismanis [18] developed an AI-based robotic solution capable of autonomously performing parcel placement tasks, providing valuable insights for the construction of automated parcel delivery systems. Zhang [19] utilized STM32F4 as the main controller, establishing a flat visual coordinate system, using a CCD image sensor, and achieved automatic sorting of parcels. Combining the above domestic and foreign research status and research results, we can draw two conclusions:

1. The introduction of visual technology into intelligent logistics sorting systems has been the main direction of research in the industry in recent years, serving as a crucial means to enhance the performance of intelligent logistics sorting systems.
2. Different visual detection methods exist for various intelligent logistics sorting systems in different industries and scenarios.

## 3. Proposed Method

### 3.1. Overall Structure Design of Image Acquisition

The intelligent logistics sorting system can be delineated into three distinct stages: entry of parcels to the sorting station (Stage 1), their transfer to the sorting trolley (Stage 2), and their subsequent departure from the trolley (Stage 3). Owing to variables like manual errors and system stability in Stage 1, parcel positioning on the sorting trolley exhibits variability during the transition from Stage 1 to Stage 2. The initiation of Stage 3 is contingent upon the parcel’s positioning on the trolley. In instances where the parcel’s position on the trolley is indeterminate, the likelihood of incorrect routing to an erroneous chute increases, potentially leading to a malfunction in the intelligent logistics sorting system.

**Figure 2:**
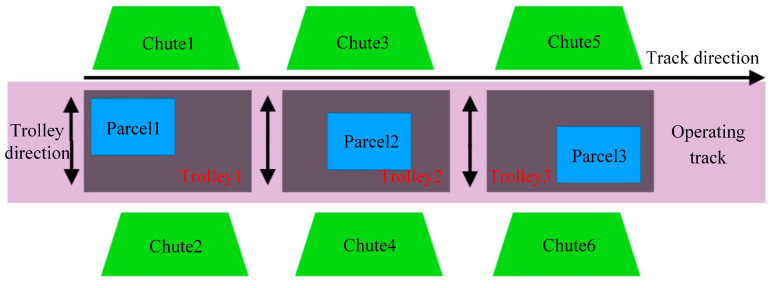
Sports package sorting diagram.

**Figure 3:**
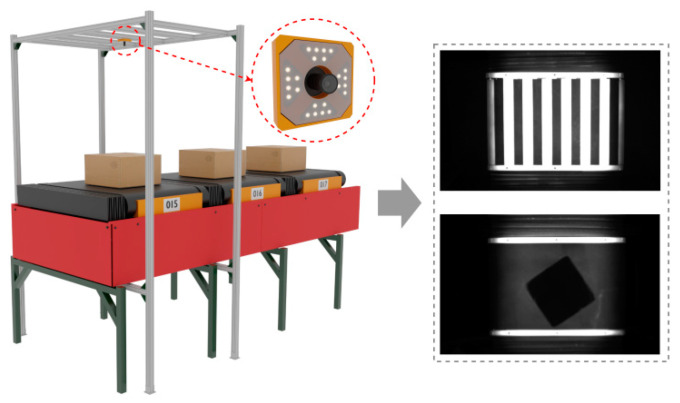
Schematic diagram of the overall structure of image acquisition.

As depicted in Figure 2, trolleys 1 to 3 are mounted on the running track and navigate along the track’s axis (left and right). Chutes 1 to 6 are strategically positioned on either side of the running track. Parcels are placed on the trolleys’ surfaces, and as the trolleys advance to the designated drop-off positions along the track, the parcels descend into the corresponding chutes, aligned with the trolley’s trajectory (up and down), thereby concluding the sorting process. Owing to the positional variations in parcels on the sorting trolleys (as demonstrated in Figure 1, Parcel 1 to Parcel 3), the precise drop-off location varies for each trolley. The drop-off location is ascertained based on the parcel’s relative position on the trolley. This study introduces a novel algorithm for motion-based parcel position correction, integrating traditional and AI vision technologies. The algorithm precisely computes the relative positioning of parcels on each trolley, establishing the foundation for determining the drop-off location for each trolley, consequently enhancing the precision of parcel sorting in intelligent logistics systems. To realize this objective, the paper delineates the comprehensive structure for image acquisition, as exemplified in Figure 3.

Above the moving track, a bracket is installed with an industrial camera based on brightness values. The lens of the industrial camera is parallel to the moving track, capturing grayscale images. As the trolley and parcel pass through the bracket, the camera records a real-time image containing information about the positions of the trolley and parcel. This image is then transmitted to the image acquisition data center of the intelligent logistics sorting system. This entire process constitutes one complete image acquisition cycle. In a real intelligent logistics sorting center, the image acquisition process will be repeated multiple times, and the conditions for each image capture will be the same. This repetition provides crucial real-time information for the subsequent recognition and sorting of parcel positions.

### 3.2. Design of Traditional Visual Package Positioning Algorithm

This study utilizes traditional vision techniques, predicated on variations in grayscale values, to facilitate the position correction of moving express parcels. This methodology entails the segmentation of the trolley’s detection area and parcel localization through traditional vision.

#### 3.2.1. Trolley Detection Area Division

**Figure 4:**
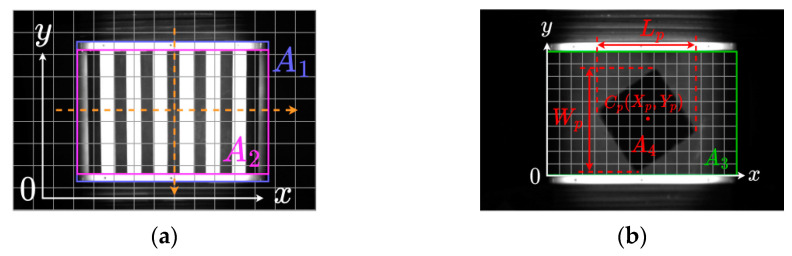
Traditional visual package positioning: (**a**) trolley positioning method and (**b**) parcel location method.

Through the image acquisition structure in Section 3.1, we can obtain multiple sets of images containing information about the trolley’s position. Selecting an image containing trolley number one serves as the basis for traditional vision to partition the trolley detection area, as shown in Figure 4a. The original image is divided into A×B small regions, and the average grayscale value of all pixels in each region is taken as the grayscale value of that region. The coordinate of the region’s center point is considered as the coordinate of that region. Column scanning and row scanning are performed on the middle part of the image, counting the coordinate information of all small regions whose grayscale values exceed the set threshold. From these, the maximum and minimum values of *x* or *y* are, respectively, selected to obtain the relative coordinate information $A_{1}=\left\{(x,y)|x_{\min}\leq x\leq x_{\max},y_{\min}\leq y\leq y_{\max}\right\}$ of region $A_{1}$ within the image area. To reduce interference from the edges of the trolley, region $A_{1}$ is cropped from the image and further divided into C×D small regions. Column scanning is performed on the middle of region $A_{1}$, counting the coordinate information of all small regions whose grayscale values are below the set threshold. From these, the maximum and minimum values of *y* are selected to obtain the relative coordinate information $A_{2}$ of region $A_{1}$ within area $A_{2}=\left\{(x,y)|0\leq x\leq 1,y_{\min}^{\prime }\leq y\leq y_{max}^{\prime }\right\}$. Finally, region $A_{2}$, cropped from region $A_{1}$, is the detected area for the trolley.

#### 3.2.2. Parcel Location

Selecting the trolley detection area, $A_{2}$, from Section 3.2.1 for package positioning, $A_{2}$ is divided into M×N small regions to obtain region $A_{3}$. Different grayscale value thresholds are set for the middle and left/right sides of region $A_{3}$. The grayscale values of all small regions in region $A_{3}$ are counted. *S* small regions with grayscale values exceeding the set threshold are considered to be the package coverage area, $A_{4}$, and the coordinate information of all small regions in region $A_{4}$ is recorded. The difference between the maximum and minimum values of *x* and *y* in the coordinate information of *s* small regions gives the relative length, $L_{p}$, and width, $W_{p}$, of the package to region $A_{3}$. The average of the *x* and *y* values in the *s* small regions is calculated, resulting in the relative coordinates $\left(X_{p},Y_{p}\right)$ of the package center $C_{p}$ to region $A_{3}$. Finally, based on the distance scaling ratio between the image and reality, the real-world coordinates of the package relative to the trolley can be determined. The overall steps are shown in the following formula:

(1) $$\begin{array}{l} L_{r}=\lambda L_{p}X=\lambda (x_{\max}-x_{\min}),W_{r}=\lambda Wp=\lambda (y_{\max}-y_{\min})\\ X_{r}=\lambda Xp=\lambda \frac{1}{s}\sum_{i=1}^{s}x_{i},Y_{r}=\lambda Y_{p}=\lambda \frac{1}{s}\sum_{i=1}^{s}y_{i} \end{array}$$

In Equation (1), $L_{r}$ and $W_{r}$ represent the actual length and width of the package; $L_{p}$ and $W_{p}$ denote the relative length and width of the package to region $A_{3}$; $x_{\max}$ and $x_{\min}$ are the maximum and minimum *x* values in the coordinate information of *s* small regions; $y_{\max}$ and $y_{\min}$ are the maximum and minimum *y* values in the coordinate information of *s* small regions; $X_{r}$ and $Y_{r}$ stand for the *x* and *y* values of the actual package center relative to the trolley; $X_{p}$ and $Y_{p}$ represent the relative *x* and *y* values of the package to region $A_{3}$; and λ is the distance scaling ratio between the image and reality.

### 3.3. Parcel Location Algorithm Design of AI Vision

This study implements a deep learning-based object detection model to correct the positioning of moving express parcels. The relative coordinates of the parcel to the trolley are determined by subtracting the coordinates of the parcel’s predicted bounding box from those of the trolley. The calculation formula is as follows:

(2) $$X_{r}=\lambda X_{p}=\lambda (x_{e}-x_{c}),Y=\lambda Y_{p}=\lambda (y_{e}-y_{c})$$

In Equation (2), $x_{e}$ and $y_{e}$ represent the coordinates of the center point of the predicted bounding box for the package, and $x_{c}$ and $y_{c}$ represent the coordinates of the center point of the predicted bounding box for the trolley.

#### 3.3.1. Algorithm Basis

**Figure 5:**
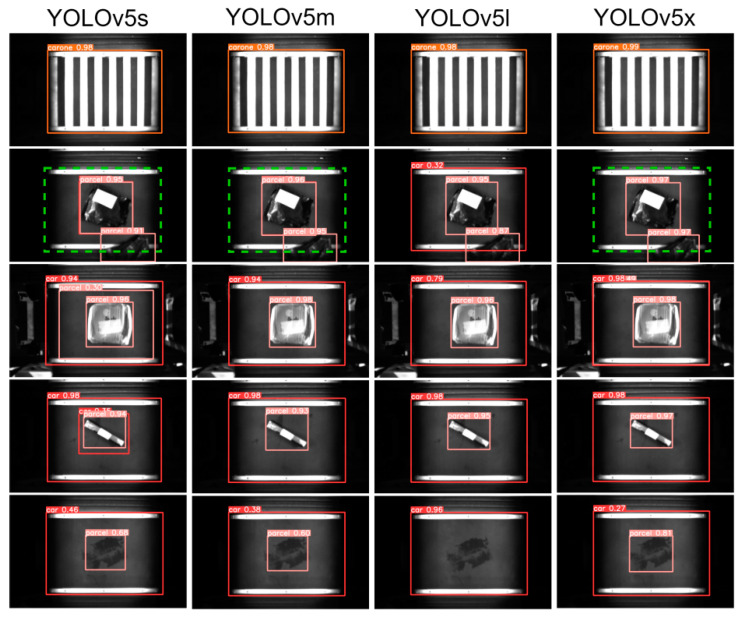
Comparison of detection accuracy. (The orange frame identifies the car one, the red frame identifies the ordinary car, and the pink frame identifies the package).

Given YOLOv5′s outstanding performance in inference speed and recognition accuracy among one-stage algorithms, this study refines YOLOv5 to develop a parcel positioning algorithm tailored for actual intelligent logistics sorting systems. We selected four commonly used variants (s,m,l,x) of YOLOv5 for experiments comparing the detection accuracy of trolleys and packages. Figure 5 and Table 1 present the experimental results.

Combining Figure 5 and Table 1 reveals that, among the four variants of YOLOv5, YOLOv5x achieves the highest precision, YOLOv5s exhibits the fastest inference speed, and YOLOv5l has the highest recall. Considering the high demand in real logistics centers for both detection accuracy and inference speed of the detection model, we chose the YOLOv5l model, which has the highest F1 score, as the baseline for the AI visual package positioning algorithm in this paper.

#### 3.3.2. Convolutional Block Attention Module

**Figure 6:**
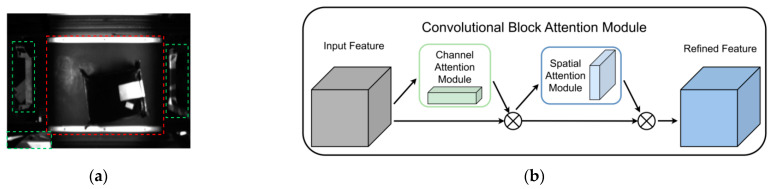
Convolutional Block Attention Mechanism: (**a**) identification and positioning situation and (**b**) modular structure. (The red frame represents the car, the green frame represents the misidentified objects).

In real logistics centers, there is often a significant amount of clutter around the conveyor belt. Therefore, we aim for the model to focus more on the information related to the trolley and the package in both the training and inference stages (as shown in the red area of Figure 6a), while reducing interference from irrelevant information (such as the green area in Figure 6a). To enhance the model’s perceptual and generalization abilities, we introduce the Convolutional Block Attention Mechanism (CBAM) [20]. CBAM is a simple yet effective attention module in feedforward convolutional neural networks, combining both channel attention and spatial attention mechanisms applied to the convolutional block, as illustrated in Figure 6b.

**Figure 7:**
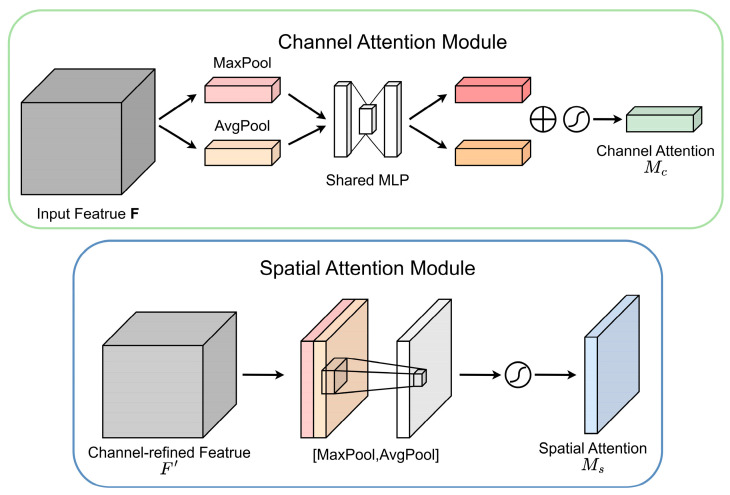
Schematic representation of channel and spatial attention mechanisms.

As illustrated in Figure 7, in the channel attention module (CAM), the input features are first subjected to maximum pooling and average pooling respectively to aggregate the spatial information of the feature map and generate corresponding spatial context features $F_{\max}^{s}$ and $F_{avg}^{c}$. Then $F_{\max}^{c}$ and $F_{avg}^{c}$ are input to a multilayer perceptron (MLP) with hidden layers, and the output elements are summed item by item. Finally, the sigmoid function is passed to generate the channel attention map $M_{c}\in R^{c\times 1\times 1}$. To reduce the number of parameters, the hidden layer activation size in multilayer perceptron (MLP) is set to $R^{c/r\times 1\times 1}$.

In the spatial attention module (SAM), the output results of the channel attention module are subjected to maximum pooling and average pooling operations to obtain two 1×H×W feature maps $F_{\max}^{s}$ and $F_{avg}^{s}$, and then the two feature maps are spliced through a splicing operation. It is converted into a 1-channel feature map through 7×7 convolution, and then a sigmoid function is used to obtain the feature map of the spatial attention module. Finally, the output result is multiplied by the original image and returned to C×H×W size.

The approximate calculation process of the overall CBAM is expressed by the following formula:

(3) $$\begin{array}{l} F^{\prime }=M_{c}(F)⊗F,\\ F^{\prime \prime }=M_{s}(F^{\prime })⊗F, \end{array}$$

In the above formula, ⊗ represents element-wise multiplication, and $F^{\prime \prime }$ represents the final output. $M_{c}$ and $M_{s}$ can be obtained by the following formula:

(4) $$\begin{array}{l} M_{c}(F)=\sigma (MLP(AvgPool(F))+MLP(MaxPool(F)))\\ \;=\sigma (W_{1}(W_{0}(F_{avg}^{c}))+W_{1}(W_{0}(F_{\max}^{c}))) \end{array}$$

(5) $$\begin{array}{l} M_{s}(F)=\sigma (f^{7\times 7}([AvgPool(F);MaxPool(F)]))\\ \;=\sigma (f^{7\times 7}([F_{avg}^{s};F_{\max}^{s}])) \end{array}$$

In the above formula, σ(⋅) represents sigmoid function, and $W_{0}\in R^{c/r\times c}$ and $W_{1}\in R^{c\times c/r}$ represents the hidden layer weights and output layer weights in multilayer perceptron (MLP).

#### 3.3.3. Focal-EIoU

**Figure 8:**
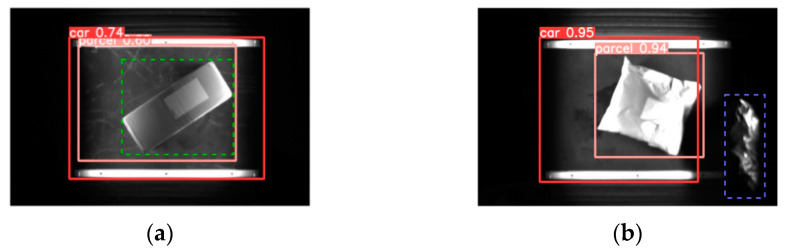
Comparison between predicted box and true box: (**a**) situations where the predicted box misses coverage and (**b**) handling of challenging samples. (The red frame identifies the car, the pink frame identifies the parcel, the green frame represents the parcel, the blue frame represents the misidentified objects.).

While the CIoU loss function [21] employed in YOLOv5 progressively approximates the width-to-height ratio of the actual bounding box, there are instances where the predicted box may not fully encompass the package, as depicted in Figure 8a. Furthermore, the model encounters difficulties in processing complex samples (illustrated in Figure 8b), resulting in occasional omissions of packages and compromised quality of the predicted bounding box for the trolley.

To improve the quality of predicted bounding boxes, this study integrates Focal-EIoU [22] as the loss function to enhance the YOLOv5 network. The Focal-EIoU loss function accounts for discrepancies in width and height between the predicted and actual bounding boxes. By directly calculating penalties for these discrepancies, it enhances the model’s convergence rate and detection precision. Furthermore, considering the imbalance of easy and challenging samples in bounding box regression within object detection, Focal-EIoU elevates the influence of challenging samples on the bounding box regression optimization, thereby bolstering the model’s generalizability. The computation of Focal-EIoU is shown in Equation (4):

(6) $$\begin{array}{l} L_{Focal-EIoU}=IoU^{\gamma }L_{EIoU}\\ L_{EIoU}=1-IoU+\frac{\rho^{2}(w,b^{gt})}{c^{2}}+\frac{\rho^{2}(w,w^{gt})}{c_{w}^{2}}+\frac{\rho^{2}(h,h^{gt})}{c_{h}^{2}} \end{array}$$

In Equation (4), γ represents the parameter controlling the degree of outlier suppression; $\rho^{2}(w,b^{gt})$ denotes the Euclidean distance between the centers of the predicted and true bounding boxes; $\rho^{2}(w,w^{gt})$ signifies the Euclidean distance between the widths of the predicted and true bounding boxes; $\rho^{2}(h,h^{gt})$ represents the Euclidean distance between the heights of the predicted and true bounding boxes; *c* represents the diagonal distance of the minimum bounding rectangle; and $C_{w}$ and $C_{h}$, respectively, represent the width and height of the minimum bounding rectangle.

#### 3.3.4. Optimal Transport Assignment

During the initial phases of training the model for moving express parcels, each ground truth (gt) generates numerous anchors. Anchors labeled for either the trolley or the parcel are classified as positive samples, whereas others are deemed negative samples. Considering the direct impact of positive and negative sample assignment on model performance, this study utilizes Optimal Transport Assignment (OTA) [23], a method grounded in a global optimization strategy. This approach frames the labeling of trolleys and parcels as an optimal transport problem, conceptualized between suppliers and demanders. Specifically, assuming there are *m* ground-truths and *n* anchors in a given input image (spanning all Feature Pyramid Network [24] layers), where the *i*th ground-truth has *k* positive sample labels $\left(i.e.,s_{i}=k,i=1,\cdot \cdot \cdot ,m\right)$, the *j*th anchor requires a positive/negative label $\left(i.e.,d_{j}=1,j=1,\cdot \cdot \cdot ,n\right)$, the cost for the *i*th ground-truth supplying the *j*th anchor is $c_{ij}$, and the total sum of supplies and demands for labels is $\left(\sum_{i=1}^{m}s_{i}=\sum_{j=1}^{m}d_{j}\right)$, the goal is to find an optimal assignment plan $\min\sum_{i}^{m}\sum_{j}^{n}C_{ij}\pi_{ij}$ that minimizes the transportation cost between all ground-truths and anchors.

Define the transmission cost of $gt_{i}$ to assign anchor $a_{j}$ a positive sample label (such as a trolley or package) as $C^{fg}\in R^{m\times n}$. An additional supplier (background) with n−m×k negative sample labels is set, and the transmission cost of a negative sample label assigned by background to anchor $a_{j}$ is defined as $C^{bg}\in R^{1\times n}$. Concatenating $C^{bg}\in R^{1\times n}$ to the last row of $C^{fg}\in R^{m\times n}$ gives the total cost matrix, $C\in R^{(m+1)\times n}$. According to the label supply vector, $S\in R^{\left(m+1\right)}$, and label demand vector, $D\in R^{n}$, the optimal positive/negative label allocation strategy, $\pi^{*}\in R^{(m+1)\times n}$, can be obtained through the Sinkhorn–Knopp Iteration [25]; that is, the sports express package detection model can obtain the total training sample based on global optimization, and each position in the feature map can obtain the most appropriate learning target. The specific formula is as follows:

(7) $$\begin{array}{l} C_{ij}^{fg}=L_{cls}(P_{j}^{cls}(\theta ),G_{i}^{cls})+\alpha L_{reg}(P_{j}^{box}(\theta ),G_{i}^{box})\\ C_{j}^{bg}=L_{cls}(P_{j}^{cls}(\theta ),\phi ) \end{array}$$

In Equation (5), θ denotes the network parameters; α represents the balance coefficient; and $P_{j}^{cls}$ and $P_{j}^{box}$ signify the predicted class score and the bounding box $a_{j}$, respectively. $G_{i}^{cls}$ and $G_{i}^{box}$ stand for the ground-truth class and the bounding box $gt_{i}$. Additionally, $L_{cls}$ and $L_{reg}$ denote the cross-entropy loss and IoU [26] loss, which can be replaced by Focal Loss [27] and GIoU [28] loss. Finally, ϕ represents the background class.

#### 3.3.5. CFO-YOLOv5

The revised structure of the YOLOv5 network is depicted in Figure 7. To augment the network’s ability to process redundant and noisy data within feature maps, CBAM was integrated into the YOLOv5 backbone. Integrating CBAM enables the model to adaptively extract and amalgamate channel and spatial information from the feature maps, thereby enhancing performance in processing parcel images with interference. Within the detection head of YOLOv5, the Focal-EIoU loss function is utilized to supersede the YOLOv5 CIoU loss function. This modification addresses the challenge of maintaining consistent aspect ratios between actual and predicted bounding boxes despite significant size discrepancies, and it notably heightens the model’s attention to challenging samples. During the early phases of model training, the Optimal Transport Assignment (OTA) is employed for a globally optimized positive/negative sample labeling strategy. Contrasted with conventional label assignment techniques, OTA obviates the necessity for manual setting of global positive/negative sample IoU thresholds. It distinguishes processing for samples across different IoU ranges, offering a globally optimal assignment of training samples for the model’s parcel recognition and localization, thereby improving the quality of model training.

## 4. Experimental Test and Result Analysis

### 4.1. Preparation of Datasets

To ascertain the effectiveness of the proposed parcel localization and detection algorithm, this study employed real-time parcel images collected via the image acquisition framework outlined in Section 3.1. The images were processed to create a valid and comprehensive dataset. For the purpose of this research, 2779 real-time parcel images were compiled to constitute the experimental dataset. The real-time images were captured using the onsite industrial camera Banner VE200G1A (Produced in USA, purchased by China branch), featuring a pixel size of 752×480. To bolster the effectiveness of model training, the open-source labeling tool LabelImg was utilized to systematically convert the images into YOLO format. Subsequently, the dataset underwent random division into training, validation, and test sets at a ratio of 3:1:1.

### 4.2. Experimental Environment and Training Treatment

To guarantee the impartiality of the experiments, all models underwent training and testing on a uniform server platform. The experiments utilized Windows 10 as the operating system, equipped with an Intel(R) Xeon Gold 6248R CPU and an NVIDIA RTX A6000 GPU. The code execution environment necessitated specific library versions: Python 3.8.17, Torch 2.0.1, and CUDA 11.8. In the model training phase, the initial learning rate was established at 0.005, the batch size was determined at 24, the momentum was fixed at 0.937, the weight decay was adjusted to 0.0005, and a total of 200 epochs were executed.

### 4.3. Model Measure

Owing to the deployment of the target detection model in actual logistics centers for real-time parcel position correction, detection accuracy and inference speed are paramount evaluation metrics. This study employs primary experimental metrics, such as precision (P), recall (Recall), and F1 score (the harmonic mean of precision and recall), for model accuracy assessment, calculated as follows:

(8) Precision=TPTP+FPRecall=TPTP+FNF1=2Precision×RecallPrecision+Recall

where TP represents true positive samples predicted as positive class, FP represents false-positive samples predicted as positive class, and FN represents false-negative samples predicted as negative class.

### 4.4. Ablation Experiment

To evaluate the impact of the proposed enhancement strategies on model performance, YOLOv5 was selected as the baseline for ablation studies, with the test set serving as the experimental dataset to assess the effects of various enhancement strategies on YOLOv5′s performance. As indicated in Table 2, Experiments 2 and 3 integrated CBAM and Focal-EIoU into YOLOv5, yielding recall rate increases of 8.6% and 3.3%, respectively. The synergistic application of both strategies resulted in a notable 11.9% enhancement in recall rate. Experiment 5 exhibited a marked improvement in recall rate with the introduction of OTA, building on Experiment 4, underscoring the substantial influence of a globally optimized label assignment strategy during training on the model’s detection precision. Consequently, all proposed enhancement methodologies in this study significantly contribute to the performance enhancement of the YOLOv5 model.

**Figure 9:**
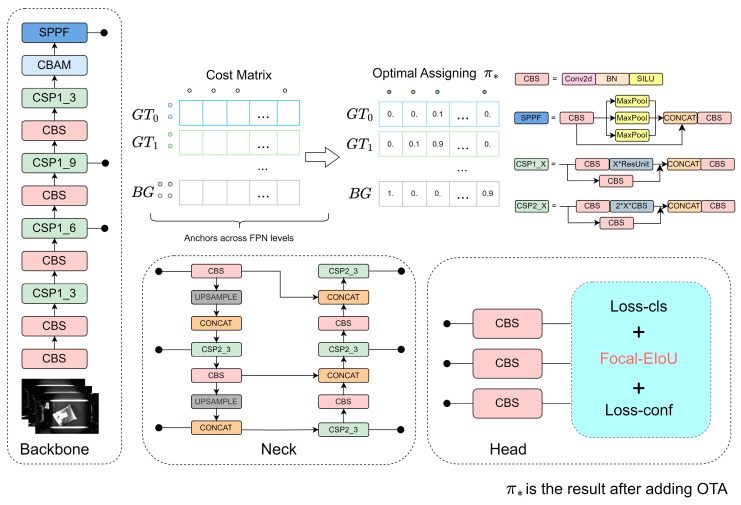
Overall structure diagram of CFOS-Yolov5.

**Figure 10:**
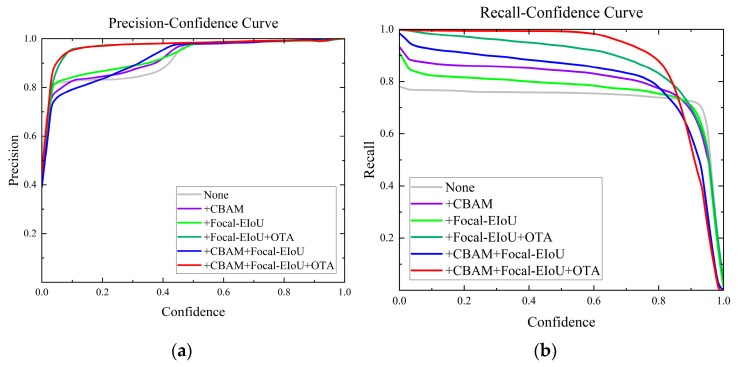

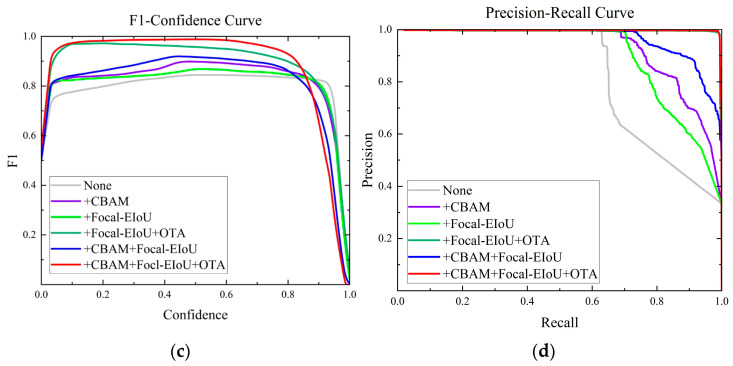
Ablation experiment model training comparison: (**a**) Precision-Confidence Curve. (**b**) Recall-Confidence Curve. (**c**) F1-Confidence Curve. (**d**) Precision-Recall Curve.

In actual logistics centers, numerous unpredictable elements, like blurred images and partially obscured parcels, can result in detection outcomes with diverse confidence levels. The objective is for the model to fulfill actual detection accuracy demands across varying confidence levels. The efficacy of various enhancement strategies at distinct confidence levels was examined and is depicted in Figure 9. As depicted in Figure 10a, OTA markedly enhances the model’s precision at lower confidence levels. Figure 10b illustrates that all proposed enhancement strategies bolster the model’s recall in the mid-to-low-confidence spectrum. Significantly, in the confidence range around 0.8, CFO-YOLOv5 demonstrates a substantial enhancement in F1 score relative to YOLOv5, as shown in Figure 10c. Figure 10d reveals that all proposed enhancement strategies lead to a considerable augmentation in the model’s mAP value.

**Figure 11:**
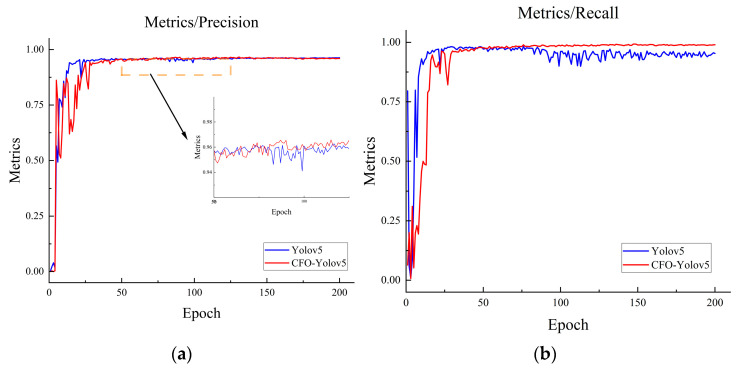
Model training comparison: (**a**) Comparison of Precision. (**b**) Comparison of Recall.

Additionally, the training process was meticulously recorded both before and after the enhancement of the YOLOv5 network. As illustrated in Figure 11, relative to the pre-enhancement YOLOv5 network, the precision and recall curves of the enhanced network demonstrate more uniform and expedited convergence throughout the training process. Notably, both curves achieve convergence approximately at the 100-epoch mark.

### 4.5. Comparative Analysis of Model Target Recognition

**Figure 12:**
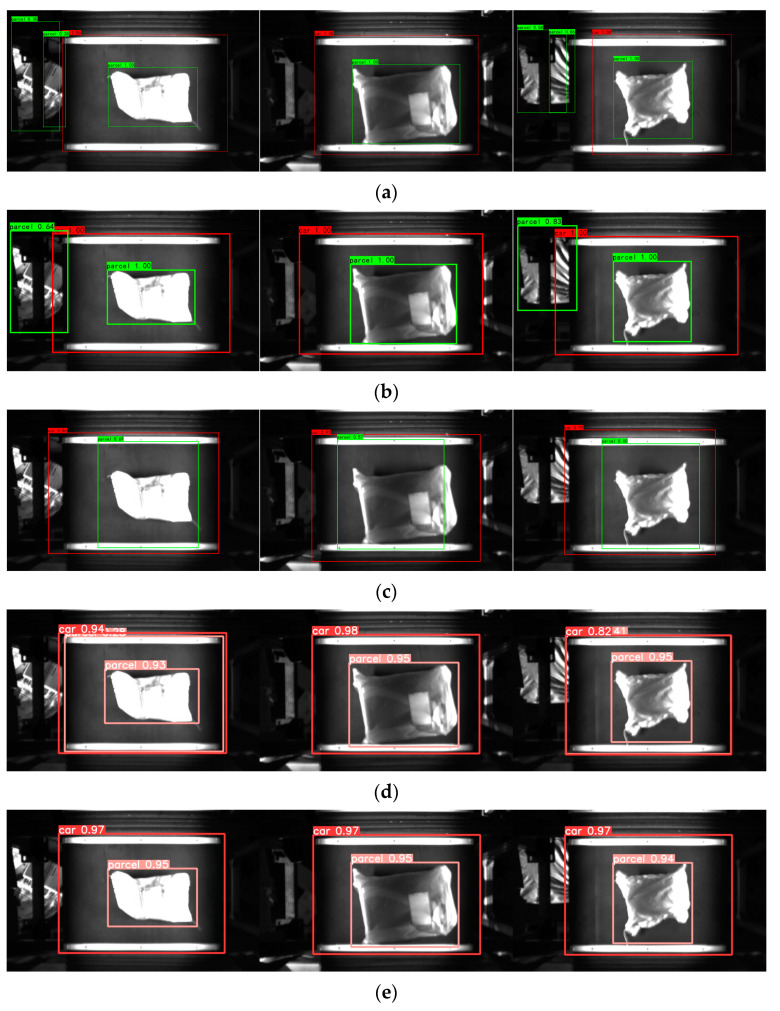
Marking results of different models for trolleys and packages: (**a**) Faster RCNN, (**b**) SSD, (**c**) RetinaNet, (**d**) Yolov5, and (**e**) ours (CFO-Yolov5).

In this research, various prevalent models in the object detection domain were chosen to compare their performance against CFO-YOLOv5, using the same test set. Table 3 showcases the performance metrics of each model, including F1, precision (P), recall (R), and frames per second (FPS), all garnered under identical experimental conditions. According to Table 3, SSD [29] exhibits the highest inference speed, whereas Faster RCNN [30] demonstrates the lowest. CFO-YOLOv5, as proposed, maintains a moderate inference speed but excels in regard to the F1 score compared to the other models. Furthermore, the study randomly selected and compared labeling outcomes of trolleys and parcels by the models in the test set, as depicted in Figure 12. Figure 12a,b reveal that both the Faster RCNN and SSD erroneously identify extraneous objects outside the trolley as parcels and display limited accuracy in trolley localization. Figure 12c indicates that while RetinaNet [27] avoided false positives for irrelevant objects outside the trolley, its accuracy in localizing trolleys and parcels is comparatively limited. Figure 12d illustrates that the original YOLOv5 incorrectly identifies a trolley as a parcel. In contrast, CFO-YOLOv5 demonstrates a relatively superior performance in this test, as shown in Figure 12e.

### 4.6. Comparison of Model Performance on Public Datasets

This study compares the proposed method with existing methodologies, utilizing the public dataset PASCAL VOC 2007. As illustrated in Table 4, the model achieves a mean Average Precision (mAP) of 69.25% on this dataset, indicating high accuracy. Furthermore, the model demonstrates superior performance in two key metrics: mAP@50 and mAP@50:95. Consequently, it can be inferred that the model exhibits robust generalization capabilities, and the proposed method is adaptable to various application scenarios.

### 4.7. Comparative Analysis of Model Target Positioning Error

**Figure 13:**
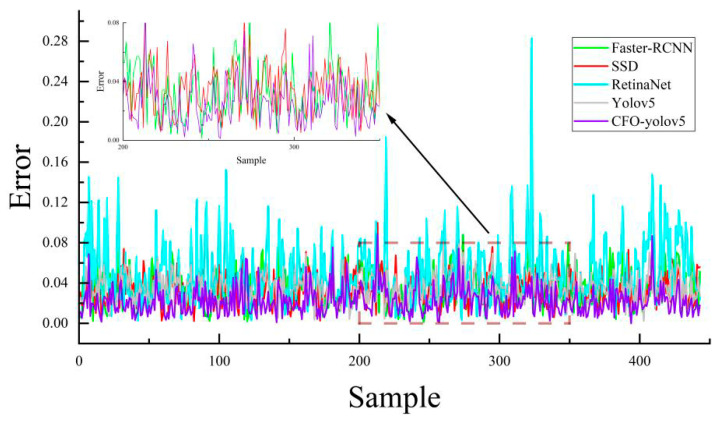
Model positioning error curve.

We selected 443 single-package images from the test set as the experimental samples to assess the model’s package localization errors. The experiment involved normalizing the target localization coordinates for different models, and the results are presented in Figure 13 and Table 5.

An analysis of Figure 13 reveals that RetinaNet experiences the most significant errors and fluctuations in package localization. YOLOv5 is next in line, exhibiting a higher error margin, while Faster RCNN and SSD demonstrate comparatively similar and lower errors in package localization. Notably, CFO-YOLOv5, as depicted in Figure 13, showcases the most impressive performance with the smallest magnitude and least fluctuation in package localization errors among the models evaluated. This superior performance of CFO-YOLOv5 is further substantiated by Table 5, which demonstrates its leading position in terms of standard deviation, mean error, and median error in package localization, thereby confirming its enhanced accuracy and reliability in this context.

### 4.8. Supplementary Experiment

When employing AI vision as the primary method for positioning moving express parcels, the goal is to achieve a more precise identification of both the trolley and the parcel, thereby minimizing the incidence of false detections of adjacent debris. This precision is vital for providing the accurate data necessary for the subsequent realignment of packages. To achieve this, the test set was utilized as the comprehensive experimental sample, and the detection outcomes of CFO-YOLOv5 for both the trolley and parcel were compiled across various confidence threshold values, as illustrated in Table 6. This approach underscores the model’s effectiveness in distinguishing relevant objects from potential background noise, which is essential for the operational success in dynamic logistics environments.

**Figure 14:**
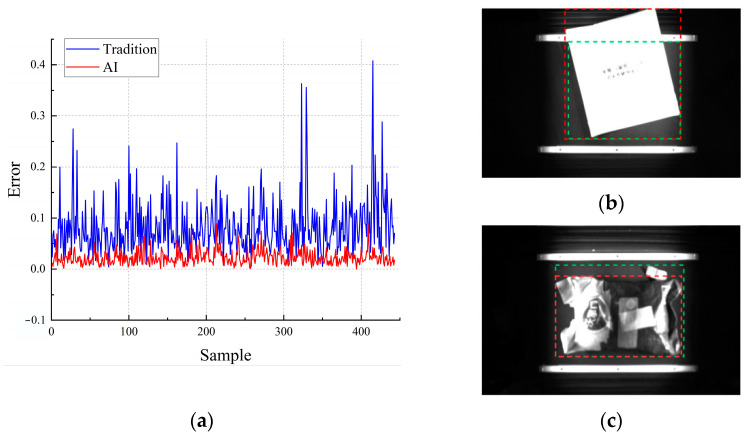
Supplementary experimental comparison: (**a**) error result normalization, (**b**) traditional visual positioning performance, and (**c**) traditional visual positioning performance. (The red frame represents the true range of parcel, the green frame represents the misrecognition range of object).

Table 6 clearly demonstrates that, as the demand for higher accuracy in identifying trolleys and packages increases, the model encounters challenges with missed package detections. Despite maintaining high precision in recognition, this study incorporated traditional vision as an auxiliary method for package positioning. To ascertain the effectiveness of integrating traditional and AI vision for express package localization, single-package precision experiments were conducted using traditional vision. CFO-YOLOv5 was employed as the benchmark for AI vision, and the single-package image dataset from Section 4.7 was used as the comprehensive experimental sample. The resulting error data were then normalized for analysis. As shown in Figure 14a, traditional vision exhibits slightly less favorable performance in terms of positioning error and fluctuation. This outcome is influenced by the intrinsic relationship between package position and shape, as illustrated in Figure 14b,c. This integration strategy highlights the strengths and limitations of both traditional and AI vision, providing insights into optimizing package localization in complex logistic environments.

Given the synergistic potential of traditional vision and AI vision in enhancing the accuracy of package positioning, the integration of traditional vision as a supplementary approach for express package localization can effectively mitigate the challenges of missed detections encountered by AI vision alone. This strategic combination leverages the strengths of both methods, offering a more robust solution for precise localization in dynamic logistic environments.

## 5. Conclusions

This paper presents an algorithm that synergizes traditional vision and AI vision for the localization of express packages on sorting robots, with the following main conclusions:

(1) The introduction of the CFO-YOLOv5 network structure for the localization of moving express packages marks a significant advancement. This enhanced structure, built upon the YOLOv5 framework, incorporates critical improvements in its backbone, head, and training sample allocation. When compared to the original YOLOv5l model, CFO-YOLOv5 registers a notable 23.6% increase in recall rate. Moreover, it surpasses classical object detection networks in both detection accuracy and inference speed.
(2) To counter the limitations of AI vision in missing detections, the paper advocates for the integration of traditional vision, particularly focusing on brightness values, as a complementary approach to express package localization. The effectiveness and practical applicability of traditional vision for this purpose were successfully validated.
(3) While the improved YOLOv5 model facilitates the rapid localization of express packages, there remains potential for further enhancements in detection accuracy and inference speed. Future work is directed towards augmenting the model’s detection capabilities, especially for uniquely shaped or special packages, to attain even higher levels of precision and efficiency.

## Figures and Tables

**Table 1:** Comparison of model detection performance.

Model	F1 (%)	Precision (%)	Recall (%)	Inference Time (ms)	mAP50 (%)
Yolov5s	85.32	98.6	75.2	10	81.9
Yolox5m	85.05	98.2	75	14	81.6
Yolov5l	85.57	98.4	75.7	19	82.3
Yolov5x	85.43	98.7	75.3	23	81.5

**Table 2:** Performance of ablation experiment.

No.	Improvement Strategy	P (%)	R (%)	F1 (%)	mAP50 (%)	mAP50:95 (%)
1	None	98.4	75.7	85.57	82.3	80.1
2	+CBAM	98	78.3	87.04	88.5	85.5
3	+Focal-EIoU	98.3	77.1	86.41	87	83.5
4	+Focal-EIoU + OTA	97.2	97.3	97.24	99.1	95
5	+CBAM + Focal-EIoU	98.1	82.2	89.45	94	89.9
6	+CBAM + Focal-EIoU + OTA	98.4	99.3	98.84	99.2	94.8

**Table 3:** Model checking performance comparison.

Model	F1 (%)	P (%)	R (%)	FPS
Faster RCNN	96.25	93.17	99.53	24
SSD	97.48	95.62	99.41	60
RetinaNet	64.73	88.59	51	28
Yolov5l	85.57	98.4	75.7	50
Ours	98.74	98.4	99.3	45

**Table 4:** Comparison of model performance on public datasets.

Model	mAP (%)	mAP@50 (%)	mAP@50:95 (%)
TinyissimoYOLO-v8 [31]	42.3%	-	-
FemtoDet [32]	22.90%	-	-
YOLOv7 + Inner-IoU	-	64.44%	38.52%
PS-KD [33]	79.7%	-	-
Perona Malik [34]	74.37%	-	-
Ours	69.25%	68.7%	43.8%

**Table 5:** Model package positioning error comparison.

Model	Standard Deviation	Average	Median
Faster RCNN	0.0476	0.0333	0.0315
SSD	0.0463	0.033	0.0314
RetinaNet	0.08	0.05	0.0422
Yolov5l	0.0476	0.0346	0.034
Ours	0.0370	0.0226	0.0201

**Table 6:** Changes in model detection performance.

Confidence Threshold	F1 (%)	P (%)	R (%)	mAP50 (%)
0.4	99.4	99.4	99.4	99.1
0.5	99.3	99.4	99.2	99.1
0.6	98.9	99.5	98.3	98.6
0.7	97.2	99.6	94.9	96.9

## Data Availability

Data will be made available upon request.

## References

[1] Qu Y., Zhao N., Zhang H. (2023). A study on digital twin technology of Human-machine integration cross-belt Sorting System. Chin. J. Mech. Eng..

[2] Ashraf M.H., Chen Y., Yalcin M.G. (2022). Minding Braess Paradox amid third-party logistics hub capacity expansion triggered by demand surge. Int. J. Prod. Econ..

[3] Kwon K., Jun S., Lee Y.-J., Choi S., Lee C. (2022). Logistics technology forecasting framework using patent analysis for technology roadmap. Sustainability.

[4] Zhang X., Liu X. (2022). A two-stage robust model for express service network design with surging demand. Eur. J. Oper. Res..

[5] Li X. (2022). SF EXPRESS Automated Robotic Sorting System Based on Machine Learning. Proceedings of the 2022 International Conference on Urban Planning and Regional Economy (UPRE 2022).

[6] Li B., Li Z., Xu Y., Tao Y., An J. (2020). Design of weak current control system for express sorting. Int. Core J. Eng..

[7] Li Z., Xu Y., Li B., Tao Y., An J. (2020). Intelligent sorting machine design applied to express industry. Int. Core J. Eng..

[8] Zou B., De Koster R., Gong Y., Xu X., Shen G. (2021). Robotic sorting systems: Performance estimation and operating policies analysis. Transp. Sci..

[9] Khir R., Erera A., Toriello A. (2023). Robust planning of sorting operations in express delivery systems. Eur. J. Oper. Res..

[10] Li Y. (2023). A Design of Robot System for Rapidly Sorting Express Carton with Mechanical Arm Based on Computer Vision Technology. Highlights Sci. Eng. Technol..

[11] Kim M., Kim Y. (2023). Parcel Classification and Positioning of Intelligent Parcel Storage System Based on YOLOv5. Appl. Sci..

[12] Xu X., Xue Z., Zhao Y. (2022). Research on an Algorithm of Express Parcel Sorting Based on Deeper Learning and Multi-Information Recognition. Sensors.

[13] Han S., Liu X., Han X., Wang G., Wu S. (2020). Visual Sorting of Express Parcels Based on Multi-Task Deep Learning. Sensors.

[14] Wu C., Duan X., Ning T. (2023). Express parcel detection based on improved faster regions with CNN features. J. Intell. Fuzzy Syst. (Prepr.).

[15] Zhao K., Wang Y., Zhu Q., Zuo Y. (2022). Intelligent Detection of Parcels Based on Improved Faster R-CNN. Appl. Sci..

[16] Ladplee N., Pimpin A., Srituravanich W., Damrongplasit N. Volumetric Measurement of Rectangular Parcel Box Using LiDAR Depth Camera for Dimensioning and 3D Bin Packing Applications. Proceedings of the 2022 IEEE International Conference on Consumer Electronics-Asia (ICCE-Asia).

[17] Duan X., Wu C., Ning T. Study of Express Package Data Processing under Specific Scenario. Proceedings of the 2022 IEEE 10th International Conference on Computer Science and Network Technology (ICCSNT).

[18] Vismanis O., Arents J., Freivalds K., Ahluwalia V., Ozols K. (2023). Robotic System for Post Office Package Handling. Appl. Sci..

[19] Zhang Y., Cheng W. (2019). Vision-based robot sorting system. Proceedings of the IOP Conference Series: Materials Science and Engineering.

[20] Woo S., Park J., Lee J.Y., Kweon I.S. Cbam: Convolutional block attention module. Proceedings of the European Conference on Computer Vision (ECCV).

[21] Zheng Z., Wang P., Liu W., Li J., Ye R., Ren D. Distance-IoU loss: Faster and better learning for bounding box regression. Proceedings of the AAAI Conference On Artificial Intelligence.

[22] Zhang Y.-F., Ren W., Zhang Z., Jia Z., Wang L., Tan T. (2022). Focal and efficient IOU loss for accurate bounding box regression. Neurocomputing.

[23] Ge Z., Liu S., Li Z., Yoshie O., Sun J. Ota: Optimal transport assignment for object detection. Proceedings of the IEEE/CVF Conference on Computer Vision and Pattern Recognition.

[24] Lin T.Y., Dollár P., Girshick R., He K., Hariharan B., Belongie S. Feature pyramid networks for object detection. Proceedings of the IEEE Conference on Computer Vision and Pattern Recognition.

[25] Cuturi M. (2013). Sinkhorn distances: Lightspeed computation of optimal transport. Adv. Neural Inf. Process. Syst..

[26] Yu J., Jiang Y., Wang Z., Cao Z., Huang T. Unitbox: An advanced object detection network. Proceedings of the 24th ACM International Conference on Multimedia.

[27] Lin T.Y., Goyal P., Girshick R., He K., Dollár P. Focal loss for dense object detection. Proceedings of the IEEE International Conference on Computer Vision.

[28] Rezatofighi H., Tsoi N., Gwak J., Sadeghian A., Reid I., Savarese S. Generalized intersection over union: A metric and a loss for bounding box regression. Proceedings of the IEEE/CVF Conference on Computer Vision and Pattern Recognition.

[29] Liu W., Anguelov D., Erhan D., Szegedy C., Reed S., Fu C.Y., Berg A.C. (2016). Ssd: Single shot multibox detector. Lecture Notes in Computer Science, Proceedings of the Computer Vision–ECCV 2016: 14th European Conference, Amsterdam, The Netherlands, 11–14 October 2016.

[30] Ren S., He K., Girshick R., Sun J. (2015). Faster r-cnn: Towards real-time object detection with region proposal networks. Adv. Neural Inf. Process. Syst..

[31] Moosmann J., Bonazzi P., Li Y., Bian S., Mayer P., Benini L., Magno M. (2023). Ultra-efficient on-device object detection on ai-integrated smart glasses with tinyissimoyolo. arXiv.

[32] Tu P., Xie X., Ling M., Yang M., Al G., Huang Y., Zheng Y. (2023). FemtoDet: An Object Detection Baseline for Energy Versus Performance Tradeoffs. arXiv.

[33] Kim K., Ji B., Yoon D., Hwang S. Self-knowledge distillation with progressive refinement of targets. Proceedings of the IEEE/CVF International Conference on Computer Vision.

[34] Mishra S., Shah A., Bansal A., Anjaria J., Choi J., Shrivastava A., Jacobs D. (2020). Learning visual representations for transfer learning by suppressing texture. arXiv.

